# Supplementary material for: Water Uptake as a Crucial Factor on the Properties of Cryogels of Gelatine Cross-Linked by Dextran Dialdehyde
Source: Gels. 2021 Sep 30;7(4):159. doi: 10.3390/gels7040159 (PMC8544549; doi:10.3390/gels7040159)
Supplement: Supplementary file 1 [file gels-07-00159-s001.zip › gels-1388826-supplementary.pdf]

Supplementary information

Water Uptake as a Crucial Factor on the Properties of Cryogels of Gelatine Cross-Linked by Dextran Dialdehyde

Natalia Volkova [1,2] and Dmitriy Berillo [1,3,*]

[1] Department of Biotechnology, Lund University, P.O. Box 124, 22 100 Lund, Sweden; natalia.volkova@ftf.lth.se
[2] Department of Solid State Physics, Lund University, Box 118, 221 00 Lund, Sweden
[3] Department of Pharmaceutical and Toxicological Chemistry, Pharmacognosy and Botany School of Pharmacy, Asfendiyarov Kazakh National Medical University, Almaty 050000, Kazakhstan
* Correspondence: berillo.d@kaznmu.kz

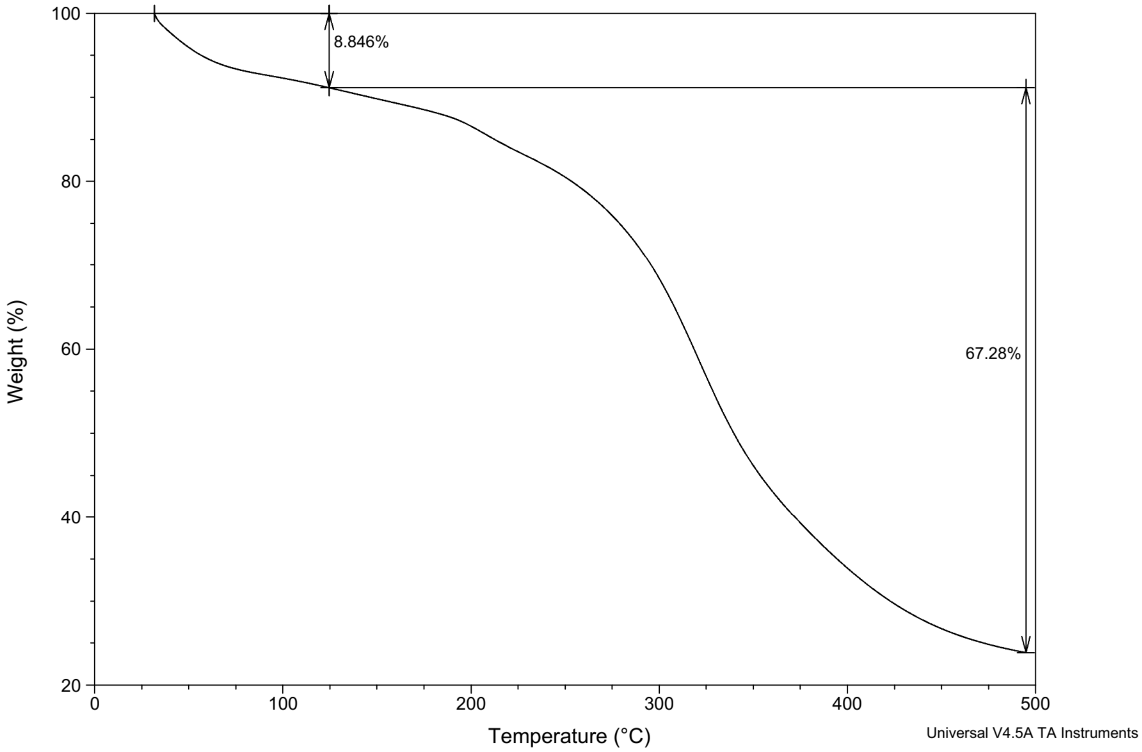

Figure S1. TGA analysis of Gel-DDA 3:1 cryogel.

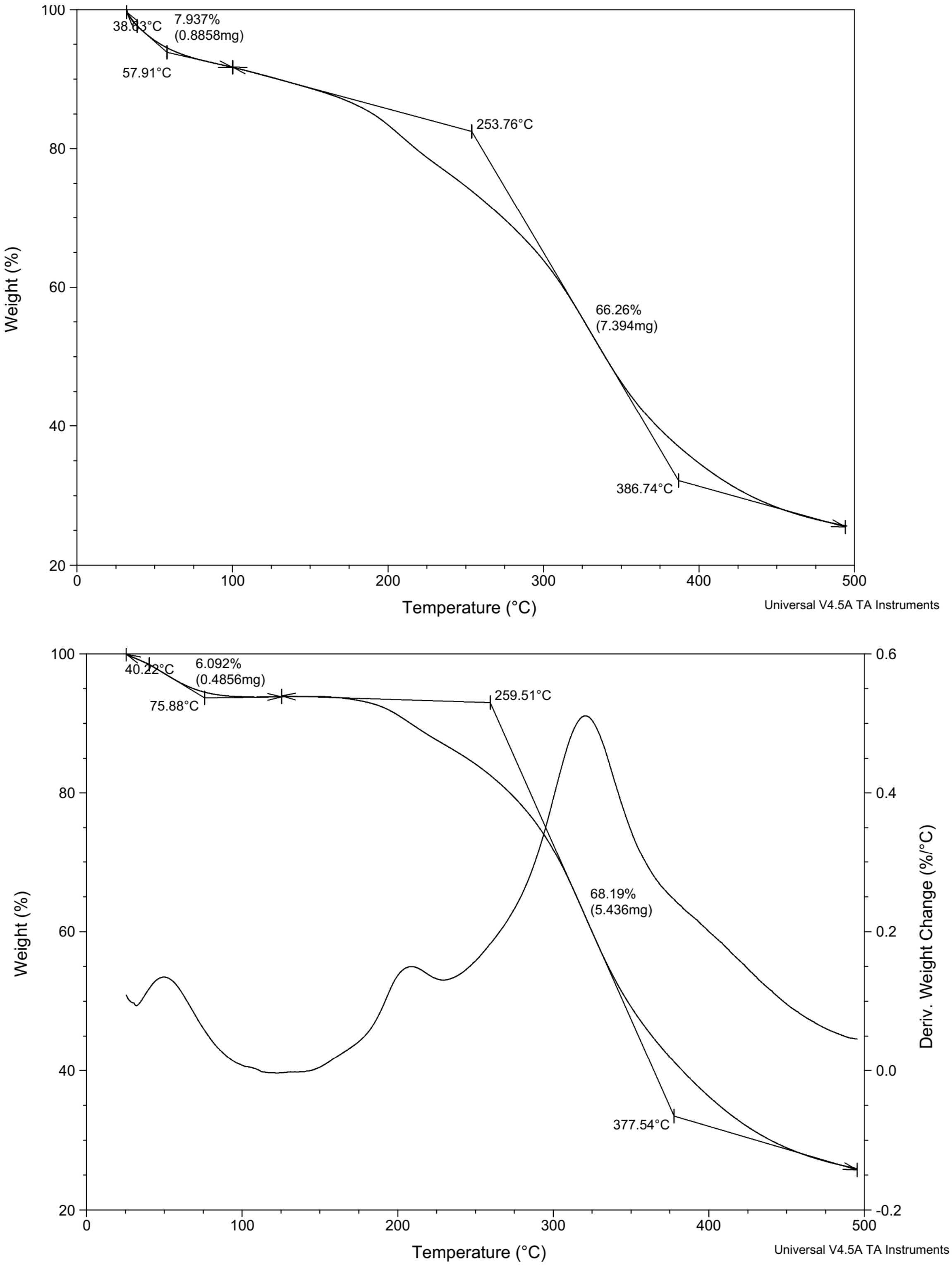

Figure S2. TGA analysis of Gel-DDA 1:1 cryogels different batches.

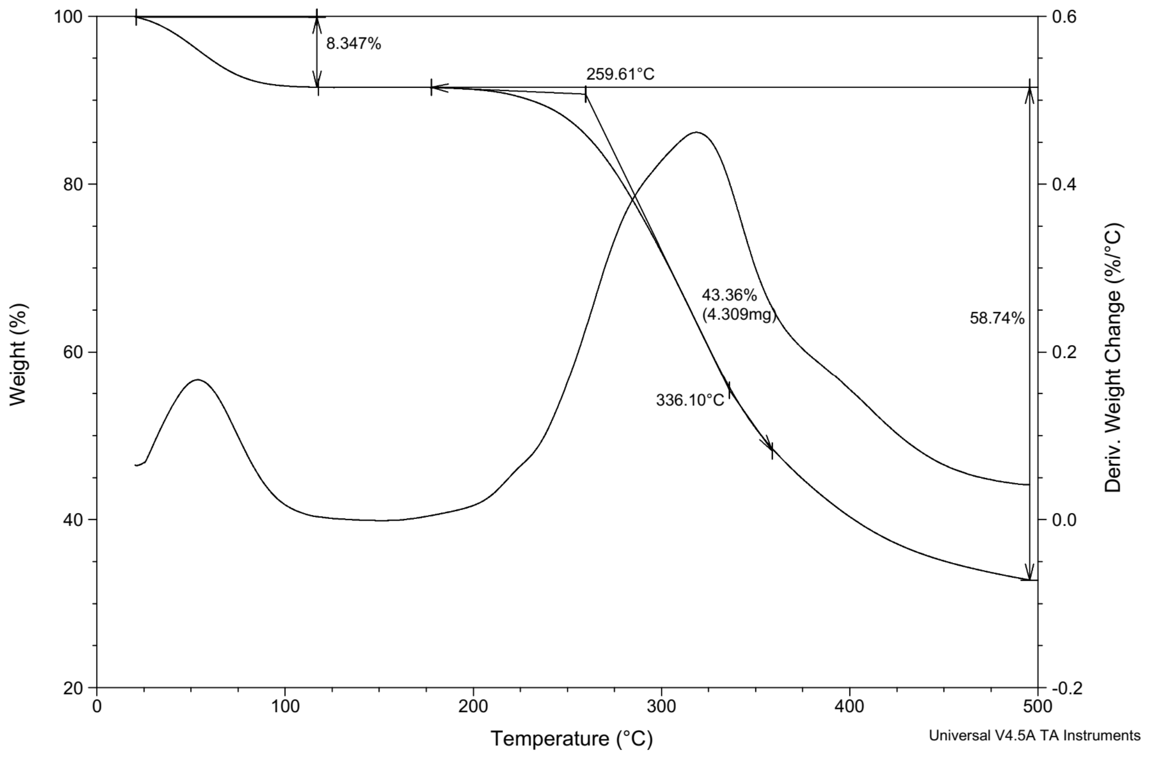

Figure S3. TGA analysis of Gel-GA 6:1 cryogel.

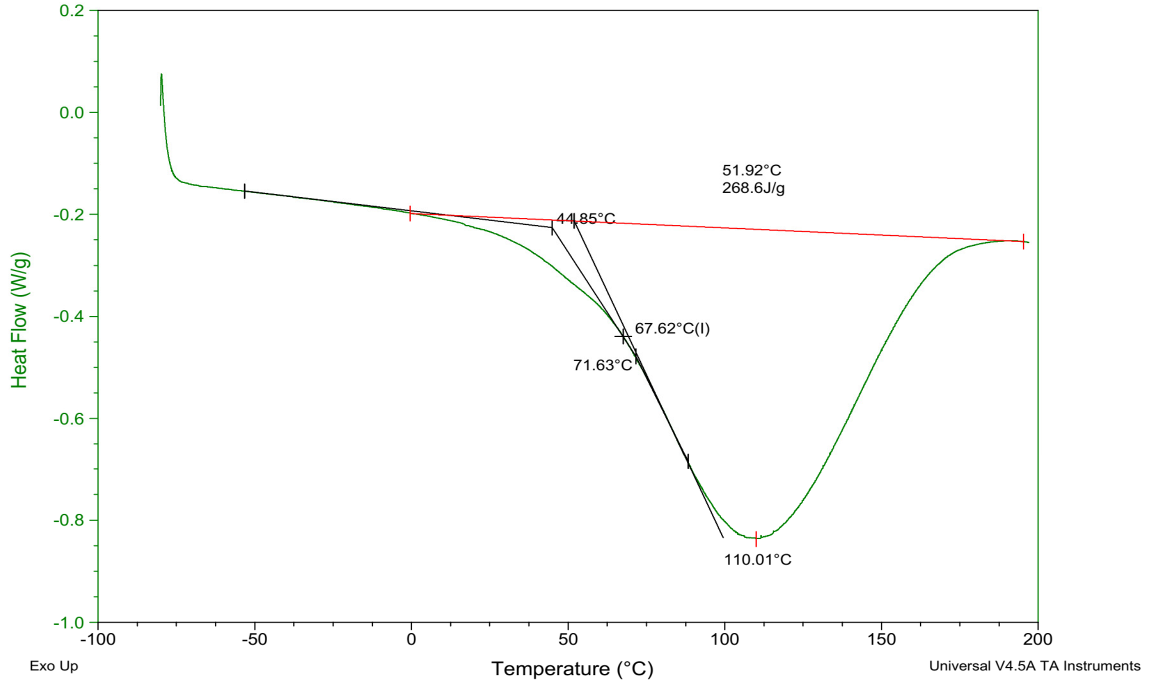

Figure S4. DSC analysis of Gel-DDA 1:1 cryogel after treatment with 100% RH and drying.

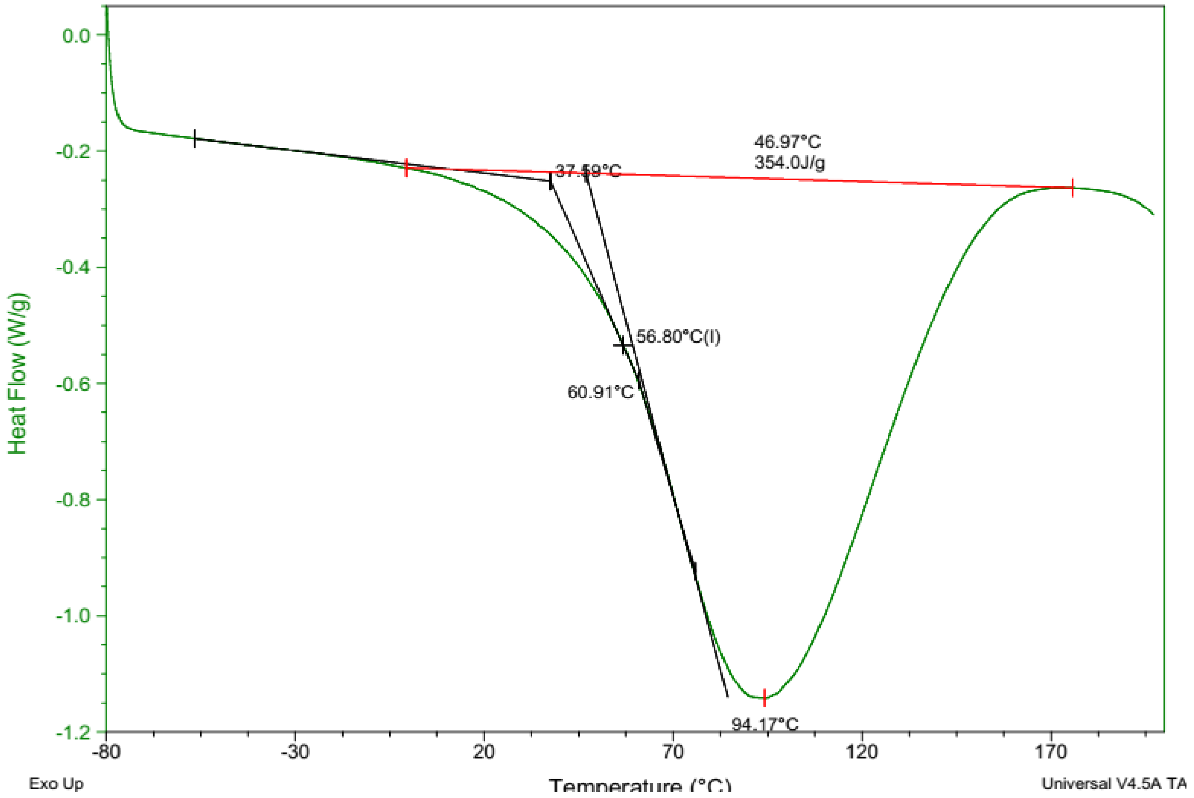

Figure S5. DSC analysis of Gel-DDA 3:1 cryogel after treatment with 100% RH and drying.

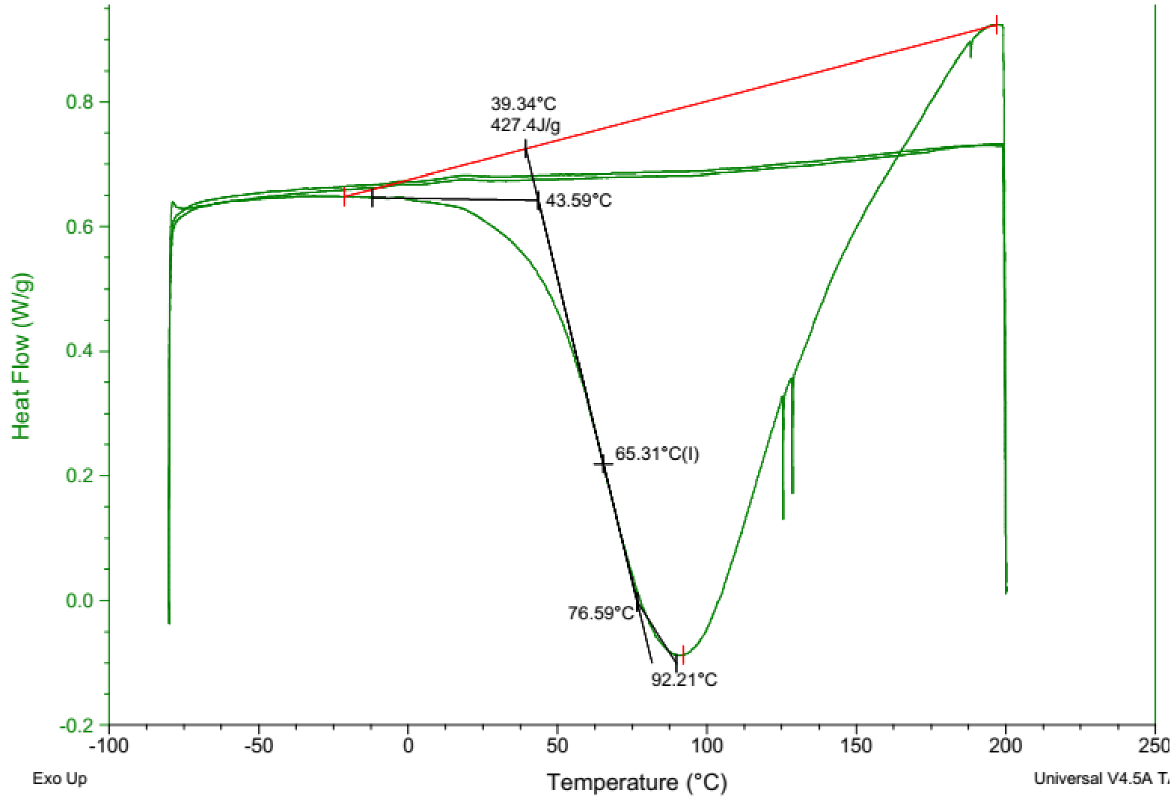

Figure S6. DSC analysis of Gel-DDA 2:1 cryogel 3 heating/cooling cycles.
